# The Impact of Intolerance of Uncertainty and Anxiety Sensitivity on Mental Health Among Public Safety Personnel: When the Uncertain is Unavoidable

**DOI:** 10.1007/s10608-020-10107-2

**Published:** 2020-04-24

**Authors:** Andréanne Angehrn, Rachel L. Krakauer, R. Nicholas Carleton

**Affiliations:** Anxiety and Illness Behaviour Laboratory, Department of Psychology, University of Regina, Regina, SK S4S 0A2 Canada

**Keywords:** Intolerance of uncertainty, Anxiety sensitivity, Mental health, Transdiagnostic, Public safety personnel

## Abstract

**Background:**

Public safety personnel (PSP; e.g., correctional workers and officers, firefighters, paramedics, police officers, public safety communications officials) are regularly exposed to potentially traumatic events and considerable uncertainty as part of their employment. Canadian PSP screen positively for mental disorders at much higher rates than the general population. Intolerance of uncertainty (IU) and anxiety sensitivity (AS) are empirically-supported vulnerability factors associated with the development and maintenance of mental disorders.

**Methods:**

The present study was designed to assess IU and AS across PSP—a population regularly encountering uncertainty—with and without mental disorders (*n* = 4304; 33.3% women), and across normative clinical, community, and undergraduate samples. Further, the study examined the relationship between IU and AS and mental disorders among PSP.

**Results:**

There were significant differences across groups on IU and AS scores (*p*s < .001). All PSP, with and without a positive screen for a mental disorder, reported lower IU and AS than clinical samples; however, PSP without mental disorders reported lower IU and AS than all other groups (*p*s < .001).

**Conclusion:**

Increased resilience or the development of coping skills to manage regular exposures to uncertain threat may help explain why PSP reported low levels of IU and AS despite higher prevalence of mental disorders. Implications for PSP training and treatment are discussed.

Public safety personnel (PSP; e.g., correctional workers and officers, firefighters, paramedics, police officers, public safety communications officials [e.g., call center operators/dispatchers]; Oliphant 2016) appear at increased risk of mental disorders given the nature of their work (Carleton et al. 2018, 2019a, b). PSP may be up to four times more likely to screen positive for at least one mental disorder than the general population (Carleton et al. 2018). PSP work may result in frequent, yet unexpected, exposure to potentially traumatic events (PTEs; Carleton et al. 2019a, b). PTEs coupled with demanding and varied work schedules make PSP vulnerable to various mental disorders such as posttraumatic stress disorder (PTSD), generalized anxiety disorder (GAD), and major depressive disorder (MDD; Benedek et al. 2007; Carleton et al. 2019a, b; Kleim and Westphal 2011). Rates of comorbidity are also cause for concern; in a sample of Canadian PSP, 18% screened positive for three or more mental disorders (Carleton et al. 2018). PSP with additional training specific to the potentially traumatic event may be less vulnerable to mental disorders in response to events and experiences encountered at work (e.g., Carleton et al. 2018; Perrin et al. 2007).

PSP work is characterized by uncertain and uncontrollable events (Barton et al. 2015; Harenčárová 2017; van den Heuvel et al. 2014). Such uncertainty may have a wide range of occupational implications for PSP who must make quick tactical decisions. For example, uncertainty may influence PSP to overestimate the negative consequences of options presented to them and result in decision avoidance (Anderson 2003; van den Heuvel et al. 2012). Uncertainty regarding work-related events may reduce overall feelings of safety among PSP, which can be associated with greater symptoms of depression and PTSD (i.e., greater hyperarousal, intrusive symptoms, peritraumatic dissociation; Fullerton et al. 2006; Grieger et al. 2004). Preliminary evidence suggests intolerance of uncertainty (IU) remains an important vulnerability factor for mental disorders among PSP (Korol et al. 2019). Accordingly, the capacity of PSP to tolerate uncertainty and manage any associated anxiety symptoms may be critical for maintaining their mental health.

IU and anxiety sensitivity (AS) have been identified as robust cognitive vulnerability factors associated with the development and maintenance of a wide range of mental disorders (Carleton et al. 2012a, b; Fedroff et al. 2000; McEvoy and Mahoney 2012; Taylor et al. 1992). The available research and theory suggest that IU and AS may also have meaningful implications for transdiagnostic prevention and treatment approaches (Carleton 2016a, b; Mahoney and McEvoy 2012). Researchers have not assessed whether levels of IU and AS among diverse PSP differ from the general population.

“IU is an individual’s dispositional incapacity to endure the aversive response triggered by the perceived absence of salient, key, or sufficient information, and sustained by the associated perception of uncertainty” (p. 31, Carleton 2016b). Individuals with high IU overestimate the possibility that a negative event will occur and perceive an inability to cope that results in maladaptive cognitive, emotional, and behavioural responses (Dugas et al. 1997, 2001). Maladaptive responses such as avoidance influence the development and maintenance of mental disorders. Elevated IU has been implicated in mood and anxiety disorders (see Carleton 2016a, for review); for example, researchers have evidenced the role of IU in depression (Carleton et al. 2012a, b; Dugas et al. 2004; Gentes and Ruscio 2011; Hong and Cheung 2015; Yook et al. 2010), social anxiety (SAD; Allan et al. 2018; Carleton et al. 2010), obsessive–compulsive disorder (OCD; Tolin et al. 2003; Gentes and Ruscio 2011), health anxiety (Boelen and Carleton 2012), panic disorder (PD; Carleton et al. 2014a, b), and posttraumatic stress disorder (PTSD; Fetzner et al. 2013). IU appears to be similarly elevated across different clinical samples and across different diagnoses; however, clinical samples as a whole have statistically significantly elevated IU when compared to community and undergraduate samples (Carleton et al. 2012a, b). IU responses (e.g., behaviour disengagement, denial, distractions) to trauma are associated with a greater likelihood of developing PTSD (Bryant and Guthrie 2007; Silver et al. 2002); as such, elevated IU may be a particularly important factor for identifying vulnerability for PSP who are regularly exposed to trauma (Carleton et al. 2019a, b). Conversely, repeated exposure to uncertainty may increase *tolerance* of uncertainty (Boswell et al. 2013b; Carleton et al. 2019a, b). Lower levels of IU consequent to such exposures may have important protective implications for PSP.

AS describes a transdiagnostic vulnerability factor based on fearing and catastrophically appraising symptoms or consequences related to anxiety (e.g., increased heart rate, perspiration; Allan et al. 2018; Boswell et al. 2013a; Carleton 2016b; Olatunji and Wolitzky-Taylor 2009; Peterson and Reiss 1987; Taylor et al. 2007), all of which inherently involve uncertainty (Carleton 2016a). PSP are regularly exposed to potentially anxiety-provoking situations (Carleton et al. 2019a, b), which implies AS may be critical for their mental health. In the general population, AS is a vulnerability factor for diverse mental disorders (Taylor et al. 2007) and is independently related to several mood and anxiety disorders (e.g., Carleton et al. 2009a, b; Fedroff et al. 2000; Taylor et al. 1992). AS has been associated with PTSD symptoms in a subset of PSP (i.e., police and firefighters) and may be an important factor in the relationship between PTSD and other mental disorder concerns (Asmundson and Stapleton 2008; Boffa et al. 2018; Stanley et al. 2017).

AS and IU are strongly correlated and seem to share common characteristics, such as the overappraisal of negative consequences and anxiety around ambiguous situations or sensations (Carleton 2016b). The two constructs do not entirely overlap (Carleton et al. 2007a, b), are differentially involved in various mental disorders (Carleton et al. 2014a, b; Fergus and Bardeen 2013; Norton et al. 2005), and may be hierarchically related (Carleton 2016b; Allan et al. 2018). Therefore, AS and IU may both be potentially efficacious targets for intervention among populations with elevated prevalence of mental disorders and comorbidities.

Despite the potential for informing PSP mental health efforts for protection and treatment (Carleton et al. in press), research regarding the role of IU and AS on PSP mental health remains limited. PSP are confronted with PTEs as well as exceptionally high levels of uncertainty as part of their occupations. Further, IU and AS appear associated with mental disorder symptoms among subgroups of PSP (i.e., police) over and above established risk factors (e.g., sex; Korol et al. 2019). Researchers do not know whether PSP differ from the general population in levels of IU and AS. Examining IU and AS among PSP may have meaningful implications for the prevention and treatment of a broad range of mental disorders. The present study was primarily designed to evaluate the role of IU and AS in the mental health of Canadian PSP. Further, the present study was designed to examine differences in IU and AS among PSP, community, and clinical samples. Based on previous research, the hypotheses included (1) PSP who experience uncertainty as part of their work would indicate lower IU and AS scores than the general population (Boswell et al. 2013a, b); and (2) among PSP, IU and AS scores would each be statistically significantly associated with mental disorder symptoms, individually accounting for variance in each measure of mental disorder symptoms.

## Methods

### Procedure

Canadian PSP were sent an online survey which assessed mental health symptoms between September 2016 and January 2017 as part of a larger study (see Carleton et al. 2018). Recruitment included emails sent to PSP agencies across Canada and the Public Safety Steering Committee (PSSC) of the Canadian Institute for Public Safety Research and Treatment (CIPSRT) and social media informing PSP of the objectives for the study and inviting participation. Interested participants completed the measures detailed below. The study was approved by the Institutional Research Ethics Board at the University of Regina (IU 2015–131).

### Participants

There were *n* = 8520 respondents who began answering the survey. A total of *n* = 4293 (33% women) respondents answered questions relating to IU and AS and were included in the current analyses. Respondents were grouped into six categories: correctional workers, firefighters, paramedics, municipal/provincial police officers, federal police officers (i.e., the Royal Canadian Mounted Police [RCMP]), and public safety communications officials (e.g., call center operators/dispatchers). An a priori power analysis conducted using G*Power (Faul et al. 2007) indicated that a minimal sample of 591 was necessary in order to achieve a medium effect size, with an alpha of .05, and a power of .80 for all planned analyses combined.

### Self-Report Measures

#### Intolerance of Uncertainty Scale, Short Form (IUS-12; Carleton et al. 2007a)

The IUS-12 is a 12-item measure designed to assess prospective IU (e.g., “Unforeseen events upset me greatly”) and inhibitory IU (e.g., “Uncertainty keeps me from living a full life”). Each item is rated on a 5-point Likert scale ranging from 1 (*not at all characteristic of me*) to 5 (*entirely characteristic of me*). Psychometric properties for the IUS-12 indicate that it is a reliable and valid measure of IU with high internal consistency (Cronbach’s α = .96), strong correlation to the original scale (*r* = .96) with both clinical and community samples (Carleton et al. 2007a; McEvoy and Mahoney 2011), and a continuous latent structure (Carleton et al. 2012c). In the current sample, the Cronbach’s was α = .92 and the average inter-item correlation was *r* = .50.

#### Anxiety Sensitivity Index-3 (ASI-3; Taylor et al. 2007)

The ASI-3 is an 18-item measure designed to assess AS (e.g., “When my chest feels tight, I get scared that I won’t be able to breathe properly”). Each item is rated on a 5-point Likert scale ranging from 0 (*very little*) to 4 (*very much*). Psychometric properties for the ASI-3 indicate that it is a reliable and valid measure of AS with high internal consistency (Cronbach’s α = .93), strong convergent, discriminant, structural, criterion validity (Taylor et al. 2007), and a taxonic latent structure (Bernstein et al. 2010). The ASI-3 has been validated in both clinical and community samples (Ghisi et al. 2016; Wheaton et al. 2012). The Cronbach’s was α = .93 for the total score in the current sample and the average inter-item correlation was *r* = .44.

#### Posttraumatic Stress Disorder Checklist for DSM-5 (PCL-5; Weathers et al. 2013)

The PCL-5 is a 20-item measure of PTSD symptoms. Items are rated on a 5-point Likert scale ranging from 0 (*not at all*) to 4 (*extremely*) while considering the burden from each symptom over the past one-month period (e.g., “How much were you bothered by repeated, disturbing dreams of the stressful experience?”). Endorsement of DSM-5 criteria for each symptom cluster and a cut-off score of 38 identified positive screens for PTSD (Carleton et al. 2018; Weathers et al., 2013). Psychometric properties for the PCL-5 indicate that it is a reliable and valid measure of PTSD symptoms, with strong internal consistency (α = .94) and test–retest reliability (*r* = .82; Blevins et al. 2015). In the current sample the internal consistency was Cronbach’s was α = .96 and the average inter-item correlation was *r* = .55.

#### Patient Health Questionnaire (PHQ-9; Kroenke et al. 2001)

The PHQ-9 is a 9-item measure of MDD symptoms. Items are rated on a 4-point Likert scale ranging from 0 (*not at all*) to 3 (*nearly every day*) while considering how often one has been bothered by each symptom over the past 2-week period (e.g., “Little interest or pleasure in doing things”). Psychometric evaluation of the PHQ-9 supports its use as a valid measure of symptoms and severity of depression, with good internal consistency (α = .89) and test–retest reliability (*r* = .84; Kroenke et al. 2001). Use of total score provides high specificity and has been recommended for screening purposes (i.e., a cut-off score of 10 indicating clinically significant symptoms; Carleton et al. 2018; Manea et al. 2015). In the current sample the internal consistency was Cronbach’s was α = 90 and the average inter-item correlation was *r* = .50.

#### Generalized Anxiety Disorder Scale (GAD-7; Spitzer et al. 2006)

The GAD-7 is a 7-item measure of generalized anxiety symptoms. Items are rated on a 4-point Likert scale ranging from 0 (*not at all*) to 3 (*nearly every day*) and assess distress over the past two weeks (e.g., “Not being able to stop or control worrying”). The GAD-7 has strong psychometric properties with high internal consistency (α = .92) and good test–retest reliability (*r* = .88; Spitzer et al. 2006). A cut-off score of 10 was used to identify positive screens for GAD (Carleton et al. 2018; Swinson 2006). In the current sample the Cronbach’s was α = .92 and the average inter-item correlation was *r* = .62.

#### Social Interaction Phobia Scale (SIPS; Carleton et al. 2009a, b)

The SIPS is a 14-item measure of social anxiety symptoms. Each item is measured on a 5-point Likert scale ranging from 0 (*not at all*) to 4 (*extremely*). The SIPS is comprised of items from the Social Interaction Anxiety and Social Phobia Scales (e.g., “When mixing socially I am uncomfortable”; Mattick and Clarke 1998). Psychometric evaluation of the SIPS indicate it is a reliable measure of social anxiety with good convergent and discriminant validity (Carleton et al. 2014b; Duranceau et al. 2014; Reilly et al. 2012). A cut-off score of 21 was used to indicate clinically significant social anxiety. In the current sample, the Cronbach’s was α = 94 and the average inter-item correlation was *r* = .50.

#### Panic Disorder Severity Scale (PDSS; Shear et al. 1997)

The PDSS is a 7-item measure of panic disorder symptoms. Each item is rated on a 5-point Likert scale ranging from 0 (e.g., *not at all*) to 4 (e.g., *nearly constantly and to a disabling extent*). Items assess the impact of symptoms overs the past week (e.g., “During the past week, how much have you worried or felt anxious about when your next panic attack would occur or about fears related to the attacks?”). A cut-off score of 7 was used to identified positive screens for PD (Shear et al. 1997). Psychometric evaluations of the measure have demonstrated good reliability (*r* = .71) and internal consistency (α = .88; Shear et al. 2001). In the current sample, the Cronbach’s was α = .93 and the average inter-item correlation was *r* = .67.

### Statistical Analyses

Descriptive statistics were calculated for IU and AS, as well as for each self-report measure of mental disorder symptoms, for the total sample, for PSP with a positive mental disorder screen, and for PSP who did not screen positive. Correlations were computed between each mental disorder assessed and IU and AS respectively. Fisher’s *r* to Z transformations (i.e., Fisher’s Z scores) were computed to assess any significant differences between mental disorder symptoms and IU and AS in each PSP category. Analysis of variance (ANOVA) with post-hoc Tukey comparisons was conducted to discern differences among PSP, community, and clinical samples on IU and AS. PSP with and without a positive screen on one or more mental disorders were compared to clinical, undergraduate, and community samples on their reports of IU (Carleton et al., 2012a, b) and to community samples on their reports of AS (Taylor et al. 2007). Linear regression analyses were used to determine if levels of IU and AS explained statistically significant variance in mental disorder symptoms (i.e., PTSD, MDD, GAD, SAD, and PD). Regression models included IU and AS independent variables, and each individual mental disorder as the dependent variable.

## Results

### Demographic and Mental Disorder Symptom Descriptive Statistics

Demographic information characterizing the sample is presented in Table 1. Most PSP were from Western Canada, had some post-secondary education, and were English speaking. Overall, 50% of PSP screened positive for at least one mental disorder. The most common mental disorders that PSP screened positive for were PTSD and GAD followed by SAD, PD, and MDD (See Table 2).

**Table 1:** Demographic characteristics among public safety personnel in Canada

	% (*n*)
Gender	
Man	66.6 (2860)
Woman	33.3 (1430)
Age	
18–29	6.0 (462)
30–39	27.5 (2122)
39–49	36.7 (2840)
50–59	25.7 (1988)
60 and older	4.1 (314)
Marital status	
Married/common-law/remarried	78.5 (3388)
Single	10.1 (437)
Separated/divorced/widowed	10.6 (459)
Ethnicity	
White	90.8 (3918)
Other	9.2 (396)
Education	
High school or less	8.8 (371)
Some postsecondary (less than 4-year college/university program)	53.6 (2253)
University degree/4-year college or higher	37.6 (1581)
Province of residence	
Western Canada (BC, AB, SK, MB)	54.1 (2296)
Eastern Canada (ON, QC)	34.6 (1470)
Atlantic Canada (PEI, NS, NB, NFL)	11.3 (478)
First language	
English	86.5 (3716)
French	10.5 (449)
Other	3.0 (128)
Public safety personnel category	
Municipal/provincial police	24.8 (1071)
RCMP	24.2 (1047)
Corrections workers	13.6 (587)
Firefighters	14.4 (623)
Paramedics	13.9 (601)
Communications Officials (e.g., all center operators/dispatchers)	4.9 (212)

*RCMP* Royal Canadian Mounted Police

**Table 2:** Mean scores on mental disorder measures and frequency of positive screens

	Mean (*SD*)			% (*n*)
	Total PSP sample	No positive screen for a mental disorder	One or more positive screen (s) for a mental disorder	
PTSD symptoms (PCL-5)	21.18 (18.74)	10.54 (9.67)	31.12 (19.42)	18.8 (806)
Depression symptoms (PHQ-9)	6.65 (5.89)	3.44 (3.15)	9.72 (6.13)	9.1 (394)
Anxiety symptoms (GAD-7)	5.29 (4.99)	2.53 (2.38)	8.01 (5.86)	18.7 (806)
Social anxiety disorder symptoms (sips)	10.46 (10.88)	5.36 (5.29)	15.08 (12.47)	15.7 (676)
Panic disorder symptoms (PDSS-SR)	2.60 (4.40)	.73 (1.54)	4.33 (4.24)	12.7 (513)

*PTSD* posttraumatic stress disorder, *PCL-5* Posttraumatic Stress Disorder Checklist for DSM-5, *PHQ-9* Patient Health Questionnaire, *GAD-7* Generalized Anxiety Disorder Scale, *SIPS* Social Interaction Phobia Scale, *PDSS-SR* Panic Disorder Symptoms Severity Scale, Self-Report, *No Dx* No mental disorder positive screen, *Dx* Any mental disorder positive screen—(i.e., posttraumatic stress disorder, anxiety, social anxiety disorder, panic disorder, and alcohol abuse) screening tools and/or who self-reported being diagnosed with a mental disorder (i.e., obsessive–compulsive disorder, persistent depressive disorder, bipolar I, bipolar II, and cyclothymic disorder)

### IU and AS Comparisons Across PSP, Community Samples, and Clinical Samples

There were significant differences with a moderate effect size across groups on IU scores *F*(5,4607) = 198.37, *p* < .001, η^2^ = .18. Post hoc Tukey analyses indicated no statistically significant differences between the previously published undergraduate sample data (Carleton et al. 2012a, b) and PSP who screened positive for one or more mental disorders (*p* = .68). All PSP, with and without a positive screen for a mental disorder, reported statistically significantly lower IU scores than previously published scores from clinical samples (MDD and GAD; *p*s < .001); however, PSP without a positive screen for one or more mental disorders reported significantly lower IU than all other comparison groups (*p*s < .001). Detailed results from the comparisons are presented in Table 3.

**Table 3:** Mean scores on intolerance of uncertainty measure across samples

Samples (*n*)	Mean (*SD*)	Tukey’s HSD comparisons mean difference (95%CI)				
		Undergrad^a^	Community^a^	MDD^a^	GAD^a^	PSP with no positive screen for a mental disorder
Undergraduate (428)^a^	27.5 (9.3)					
Community (571)^a^	29.5 (10.9)	2.0* (.4 to 3.6)				
Clinical						
MDD (26)^a^	43.0 (9.2)	15.5** (10.4 to 20.7)	13.5** (8.4 to 18.6)			
GAD (63)^a^	40.4 (11.3)	12.9** (9.4 to 16.3)	10.9** (7.5 to 14.2)	− 2.7 (− 8.6 to 3.3)		
PSP						
No mental disorder positive screens (1758)	21.1 (6.6)	− 6.4** (− 7.8 to – 5.0)	− 8.4** (− 9.6 to − 7.2)	− 21.9** (− 26.9 to − 16.9)	− 19.3** (− 22.5 to − 15.9)	
≥ 1 positive screen(s) for a mental disorder (1762)	28.2 (9.9)	.7 (− .07 to 2.1)	− 1.3* (− 2.5 to − .01)	− 14.8** (− 19.8 to − 9.8)	− 12.2** (− 15.4 to − 8.9)	7.1** (6.2 to 7.9)

*MDD* Major Depressive Disorder, *GAD* General Anxiety Disorder, *PSP* Public Safety Personnel, *No Dx* No mental disorder positive screen, *Dx* Any mental disorder positive screen—(i.e., posttraumatic stress disorder, anxiety, social anxiety disorder, panic disorder, and alcohol abuse) screening tools and/or who self-reported being diagnosed with a mental disorder (i.e., obsessive–compulsive disorder, persistent depressive disorder, bipolar I, bipolar II, and cyclothymic disorder)

**p < .001; *p < .05

^a^Samples obtained from Carleton et al. (2012a, b)

There were statistically significant differences with a moderate effect size across groups on AS scores *F*(4,8399) = 332.35, *p* < .001, η^2^ = .14. Post hoc Tukey analyses indicated that PSP without a positive screen reported statistically significantly (*p*s < .001) lower AS than previously published scores from other comparison groups (Taylor et al. 2007). PSP with a positive screen for one or more mental disorders reported significantly lower AS than clinical samples (GAD and PD; *p*s < .001), but significantly higher AS than community samples (*p* < .001). Detailed results from the comparisons are presented in Table 4.

**Table 4:** Mean scores on anxiety sensitivity measure across samples

Samples (*n*)	Mean (*SD*)	Tukey’s HSD comparisons mean difference (95%CI)			
		Community^a^	GAD^a^	PD^a^	PSP with no positive screen for a mental disorder
Community (571)^a^	12.8 (10.6)				
Clinical					
GAD (30)^a^	27.5 (16.5)	14.7** (9.1 to 20.3)			
PD (120)^a^	32.6 (14.3)	19.8** (17.0 to 22.6)	5.1 (1.1 to 11.3)		
PSP					
No positive screen for a mental disorder (1764)	8.76 (7.98)	− 4.0** (− 4.8 to − 3.2)	− 18.7* (− 24.3 to − 13.1)	− 23.8** (− 26.7 to − 21.0)	
≥ 1 mental disorder positive screens (1766)	19.97 (14.40)	7.2** (6.3 to 8.0)	− 7.5** (− 13.1 to − 1.9)	− 12.6** (− 15.5 to − 9.8)	11.2** (10.2 to 12.2)

*MDD* Major Depressive Disorder, *GAD* General Anxiety Disorder, *PSP* Public Safety Personnel, *No Dx* No mental disorder positive screen, *Dx* Any mental disorder positive screen—(i.e., posttraumatic stress disorder, anxiety, social anxiety disorder, panic disorder, and alcohol abuse) screening tools and/or who self-reported being diagnosed with a mental disorder (i.e., obsessive–compulsive disorder, persistent depressive disorder, bipolar I, bipolar II, and cyclothymic disorder)

**p < .001; *p < .05

^a^samples obtained from Taylor et al. (2007)

### Association between IU, AS, and Mental Disorder Symptoms in PSP

There was a statistically significant association between IU and AS and all mental disorder symptoms assessed. Levels of IU and AS accounted for more than 30% of variance in symptoms of PTSD, MDD, GAD SAD, and PD. Across all disorders, AS had a slightly more robust effect than IU. Detailed results are presented in Table 5. Analyses were replicated with the subfactors of IU (i.e., prospective, inhibitory) and AS (i.e., physical, cognitive, social), which were also significantly associated with all mental disorder symptom measures (all *p*s < .05). IU and AS were significantly correlated with total symptoms for each mental disorder assessed across total sample and each PSP category (*r*s = .402–.657; *p*s = .001). Correlation comparisons produced statistically significant differences across PSP categories as indicated by superscripts in Table 6. The correlations between IU and symptom measures of MDD, SAD, and PD were statistically significantly greater for RCMP when compared to other PSP groups (i.e., communications officials, firefighters, paramedics). The correlations between AS and most mental disorder symptom measures (i.e., PTSD, MDD, SAD, PD) were generally statistically significantly greater for RCMP when compared to other PSP groups.

**Table 5:** Linear regression results for variance accounted for in mental disorder symptoms by intolerance of uncertainty and anxiety sensitivity

	*B*	S.E	β	*t*	*F*	*df*	*R*^2^
PTSD symptoms					1004.24**	4222	.322
IU	.490	.033	.242	14.63**			
AS	.543	.023	.383	23.10**			
MDD symptoms					1300.32**	4264	.379
IU	.165	.010	.259	16.47**			
AS	.186	.007	.416	26.43**			
GAD symptoms					1665.72**	4264	.439
IU	.180	.008	.335	22.36**			
AS	.150	.006	.396	26.43**			
SAD symptoms					1476.13**	4268	.409
IU	.411	.018	.349	22.76**			
AS	.295	.013	.357	23.27**			
PD symptoms					1129.92**	4004	.360
IU	.075	.008	.159	9.61**			
AS	.161	.005	.486	29.36**			

*PTSD* posttraumatic stress disorder, *MDD* major depressive disorder, *GAD* general anxiety disorder, *SAD* social anxiety disorder, *PD* panic disorder, *IU* intolerance of uncertainty, *AS* anxiety sensitivity

**p ≤ .001

**Table 6:** Correlations between mental disorder symptom and intolerance of uncertainty and anxiety sensitivity by public safety personnel group

	Communications officials	Correctional workers	Firefighters	Paramedics	Municipal police	RCMP
	*r*					
PTSD symptoms						
IU	.424**	.402**	.464**	.480**	.466**	.538**
AS	.549**	.484**^a^	.561**	.500**^b^	.575**^c^	.580**^abc^
MDD symptoms						
IU	.503**	.493**	.483**^a^	.472**^b^	.525**	.561**^ab^
AS	.621**	.593**	.590**	.516**^a^	.525**^b^	.621**^ab^
GAD symptoms						
IU	.591**	.570**	.568**	.526**	.553**	.634**
AS	.643**	.657**	.588**	.568**	.575**	.625**
SAD symptoms						
IU	.491**^a^	.550**	.516**^b^	.556**	.566**	.615**^ab^
AS	.651**^ab^	.568**^c^	.465**^bcde^	.548**^df^	.561**^fg^	.628**^aefg^
PD symptoms						
IU	.481**	.437**	.440**	.405**^a^	.467**	.517**^a^
AS	.535**	.593**	.567**	.544**^a^	.569**^b^	.627**^ab^

*PTSD* posttraumatic stress disorder, *MDD* major depressive disorder, *GAD* general anxiety disorder, *SAD* social anxiety disorder, *PD* panic disorder, *IU* intolerance of uncertainty, *AS* anxiety sensitivity, *RCMP* Royal Canadian Mounted Police

**p ≤ .001

^a–g^Letter indicates a statistically significant difference between PSP groups on correlation coefficients (Fisher’s *Z* > 1.96). No letter indicates no statistically significant difference

## Discussion

IU and AS appear to be important vulnerability factors for diverse mental health disorders and have been associated with treatment outcomes (McEyoy and Mahoney 2012; Smits et al. 2008; Talkovsky and Norton 2016). Individuals with mental disorders typically endorse heightened levels of IU compared to nonclinical samples (Carleton et al. 2012a, b; Carleton 2016b), so we would expect to see heightened levels of IU among PSP with mental disorders as well. Research suggests that increased exposure to the unknown and feared situation or sensation results in reductions in IU and AS (Boswell et al. 2013a, b; Carleton et al. 2019a, b; Keough and Schmidt 2012).

The present study was designed to test the hypothesis that PSP, who experience high levels of uncertainty at work (Harenčárová 2017), will report lower IU and AS than the general population. PSP without a positive screen for one or more mental disorders reported levels of IU and AS significantly lower than community and undergraduate samples. Further, PSP with a positive screen for a mental disorder reported levels of IU comparable to community samples. The levels of IU observed in the current sample of PSP suggest a unique phenomenon contrasting previous study results in which individuals reporting clinically significant symptoms of one or more mental disorders had higher levels of IU than community sample (Carleton et al. 2012a, b; Carleton 2016b). All PSP in the current sample, with and without a positive screen for a mental disorder, reported levels of IU and AS that were significantly lower than clinical samples. Individuals with low IU report a higher capacity for tolerating absent information (Carleton 2016b); as such, low IU in PSP may be due to training received or coping skills developed to manage pervasive exposures to uncertain threat.

IU and AS have been identified as important and robust factors in the development and maintenance of mood and anxiety disorders, as well as PTSD (Carleton et al. 2012a, b; Fedroff et al. 2000; Fetzner et al. 2013; McEvoy and Mahoney 2012; Taylor et al. 1992). Accordingly, the second hypothesis examined how IU and AS were related to mental disorder symptoms in the current sample of PSP. We expected that the association between the cognitive vulnerability factors (i.e., IU, AS) and mental disorder symptoms would persist despite potential low levels of IU and AS reported by PSP. Consistent with previous results (Asmundson and Stapleton 2008; Gentes and Ruscio 2011), there was a statistically significant and robust association between IU and AS and all mental disorder symptoms (i.e., GAD, SAD, PD. MDD, PTSD). Together, IU and AS accounted for a third or more of the variance in each individual disorder. Across disorders, AS had a slightly more robust effect. The results further support postulates that IU and AS are correlated yet unique transdiagnostic vulnerability factors for anxiety, trauma and mood-related disorders (Carleton et al. 2009a, b). Further, all mental disorders screened for in the current study were statistically significantly correlated with IU and AS across all PSP, with and without a positive screen for a mental disorder; however, there were some significant differences across PSP categories. Specifically, RCMP generally had the most robust correlation relative to other PSP categories. Previously, researchers have indicated that RCMP are more likely to be diagnosed with a mental disorder relative to other PSP categories (e.g., municipal police; Carleton et al. 2018). Assessing the relationship between IU, AS, and mental disorders across each individual PSP category was beyond the scope of the current research; nevertheless, there are nuances across PSP categories that may have clinical implications. Future research should further explore the role of both cognitive risk factors in the mental health of each PSP occupation, which could play an important role in prevention and treatment strategies.

PSP reported significantly lower IU and AS than clinical samples; still, there remained a robust positive association between levels of IU and AS and mental disorder symptoms. Supporting a cognitive-behavioural treatment model, the regular exposure to uncertainty experienced by PSP appears associated with lower IU and AS (Boswell et al. 2013a, b). The current results suggest IU and AS remain clinically meaningful for PSP, despite reporting lower levels than in the clinical comparison group. Treatments for PSP may require even more poignant or tailored interventions to address IU (e.g., to uncertainty) and AS (e.g., interoceptive exposures) given the routine exposures to uncertainty and potentially difficult experiences related to AS (e.g., heart palpitations) resulting in relatively lower levels of IU and AS for PSP. However, while PSP experience routine exposures to the unknown as part of their occupation, there may be important nuances due to the uncontrolled nature of these exposures. Providing PSP with therapeutic skills (e.g., coping strategies, cognitive restructuring) to manage the psychological implications of regular encounters with the unknown may be paramount in supporting their mental health (Banducci et al. 2016). Therefore, treatment may benefit from targeting both fear of anxiety-related sensations (i.e., AS) and fear of the unknown (i.e., IU) to reduce avoidance behaviours and therein problematic mental health symptoms (Keough and Schmidt 2012; Norr et al. 2013).

There are several limitations to the present study that provide directions for future research. First, mental disorder symptoms were assessed through the use of self-report screening tools. Half of the assessed PSP screened positive for at least one mental disorder, but there is no way to know how many positive screens would ultimately be associated with a formal diagnosis. Future research should replicate the present study using clinician-administered diagnostic assessments. Second, the present data is cross-sectional; therefore, there is no way to determine whether the PSP occupational environment results in lower IU or whether individuals opting for careers as PSP have lower levels of IU. Given the distinctiveness of PSP on their levels of IU and AS, future researchers should explore whether tailored interventions targeting uncertainty result in reductions in mental health symptoms. Longitudinal research evaluating recruits throughout their careers and research evaluating PSP across treatments may help determine how to best target difficulties with uncertainty and AS. Third, the present study did not take into account all potential confounding factors in the relationship between IU, AS, and mental disorder symptoms. For example, the present study did not account for factors that have demonstrated support to be independent from IU and AS, such as negative and positive affect, neuroticism, and fear of negative evaluation (Carleton et al. 2010). The current research was also designed to examine the relationships between IU, AS, and mental disorder symptoms, rather than to address the interplay between IU and AS. Future research should specifically assess the interplay between the two cognitive risk factors in order to better understand their role in the mental health of PSP. Fourth, the present study did not examine the relationship of IU and AS to mental disorders within each PSP category. The current results suggest that both cognitive risk factors are distinctly correlated to each mental disorder assessed, but how the current results may translate into clinical divergences across PSP occupations remains unclear. While researchers have established the important role of IU and AS in the mental health of police officers (Korol et al. 2019), future research should assess the role of both cognitive risk factors in other PSP categories.

The present study results indicated that PSP with and without positive screens for a mental disorder have levels of IU and AS significantly lower than clinical samples. IU and AS remained statistically significantly associated with mental disorder symptoms. The clinical relevance of IU and AS among PSP warrants careful consideration in treatment settings and may require impactful exposures that target uncertainty.
